# Validation of a Short Form for Health Literacy Assessment Using Talking Touchscreen Technology

**DOI:** 10.3928/24748307-20200909-01

**Published:** 2020-10-08

**Authors:** Noël C. Slesinger, Kathleen J. Yost, Seung W. Choi, Elizabeth A. Hahn

## Abstract

**Background:**

Health literacy is an area of growing research and clinical interest, necessitating short, accurate measures of this complex construct. Health Literacy Assessment Using Talking Touchscreen Technology (Health LiTT) measures prose, document, and quantitative literacy by self-administration on a touchscreen computer.

**Objective:**

The objective of this study was to assess the validity of a short form of Health LiTT and to identify a meaningful cutoff score for adequate health literacy.

**Methods:**

A subsample of 137 participants from the Literacy and Cognitive Function among Older Adults study completed a 10-item Health LiTT short form and three interviewer-administered health literacy measures: Test of Functional Health Literacy in Adults (TOFHLA), Rapid Estimate of Adult Literacy in Medicine (REALM), and Newest Vital Sign (NVS). Convergent validity was assessed by correlating scores for all measures, and known-groups validity was assessed by comparing mean Health LITT scores across TOFHLA levels (inadequate, marginal, adequate). Internal consistency reliability was estimated with Cronbach's alpha. A cutoff score for adequate health literacy was established using the TOFHLA cutoff for adequate versus inadequate/marginal health literacy.

**Key Results:**

Spearman correlations between Health LiTT scores and total TOFHLA, REALM, and NVS scores were 0.65, 0.69, and 0.56, respectively (all *p* < .001). Mean Health LiTT scores were significantly and meaningfully different across inadequate (40.4), marginal (50.1), and adequate (57.1) TOFHLA categories (F = 60.6; *p* < .001). Cronbach's alpha for the Health LiTT short form was .73. A cutoff score of 55 on Health LiTT showed acceptable sensitivity and specificity to identify adequate health literacy.

**Conclusions:**

This 10-item Health LiTT short form demonstrated excellent convergent and known-groups validity and acceptable internal consistency reliability in older adults. The established cutoff also showed excellent sensitivity and specificity. Validation of other custom Health LiTT short forms with varying items from the bank and computer adaptive test-generated Health LiTT scores is ongoing. **[*HLRP: Health Literacy Research and Practice*. 2020;4(4):e200–e207.]**

**Plain Language Summary:**

This article provides evidence of the need for and psychometric properties of a valid and reliable short form of the flexible, technologically advanced Health Literacy Assessment Using Talking Touchscreen Technology measure, as well as a cutoff score to note adequate versus marginal/inadequate health literacy.

Only 12% of adults in the United States possess adequate health literacy, making it challenging for the majority of people to complete symptom questionnaires, explain symptomatology to health care providers, and fully understand health care-related materials, such as calculating medication dosage (Kutner et al., 2006). Patients with inadequate health literacy are more likely to miss preventive service appointments, have poorer health status, higher rates of hospitalization and mortality, and show poorer understanding of their medical conditions and treatments, leading to higher overall health care costs (Baker et al., 2002b, 2004; Bennett et al., 2009; Fabbri et al., 2018; Howard et al., 2005; Howard et al., 2006; Schillinger et al., 2003; Scott et al., 2002; Williams et al., 1998). Furthermore, with the increasing use of patient-reported measures and the practice of encouraging patient collaboration in health care decisions, failing to account for low health literacy may lead to inaccurate interpretation of patient-reported data and less than optimal patient engagement in patient-centered care (Carman et al., 2013; Cella et al., 2010; Elwyn et al., 2006). An accurate and concise method of measuring health literacy would be useful in both research and clinical initiatives.

Most measures of health literacy present real-world health information to assess the patient's ability to interpret and use the information objectively. These measures generally assess prose literacy (the comprehension and use of information from texts); document literacy (locating and using information from forms, tables, and graphs); or measures of quantitative literacy (applying arithmetic operations using numbers embedded in printed materials) (Kirsch et al., 1993; Yost et al., 2009) using a preset battery of questions via paper and pencil or interviewer-administered questionnaire. Health Literacy Assessment using Talking Touchscreen Technology (Health LiTT) was created to provide a more flexible, technologically advanced assessment of health literacy.

The development of Health LiTT was based on item response theory (IRT) methods that describe the underlying relationship between an overarching construct, such as health literacy, and the probability of a particular item response using carefully calibrated questions to help describe, quantify, and refine the underlying construct. Thus, Health LiTT involved the creation and calibration of a bilingual (English and Spanish) 82-item bank in a diverse sample of 608 primary care patients (Hahn et al., 2011). Health LiTT is a self-administered, multimedia (sound, images, text) tool for measuring health literacy (Hahn et al., 2011) using prose, document, and quantitative items (Yost et al., 2009; Yost et al., 2010). Additionally, document and quantitative items are accompanied by an audio recording of the question to mitigate the influence of reading comprehension on measuring those skills. Questions cover a broad range of topics relevant to primary care patients and their providers, including disease and health-related topics; Medicare, Medicaid, and insurance-related topics; and informed consent and HIPAA (Health Insurance Portability and Accountability Act)-related topics. Analyses demonstrated that the Health LiTT item bank has sound psychometric properties (Hahn et al., 2011) and although Health LiTT addresses three skills (prose, document, and numeracy), all items measured one underlying unidimensional construct of health literacy, meaning one overall score, rather than three separate scale scores, can be reported.

Providing measurement options to investigators interested in assessing health literacy facilitates the use of these tools in a variety of settings and for diverse research needs. The Health LiTT item bank supports computer adaptive testing (CAT) providing for greater opportunities for administration, such as shorter tests tailored to each person (Hahn et al., 2011), as well static short forms for quick and reliable assessments of health literacy. For example, rather than administering a CAT that results in different items being administered to each person, some clinicians and researchers, including regulatory authorities, tend to prefer static short forms because it guarantees that all respondents answer the same questions (Cella et al., 2019). In addition, researchers may want a rapid literacy assessment in their studies, and clinicians and health educators often require rapid literacy tests to identify high-risk patients. The calibrated Health LiTT item bank supports informed creation of customized short forms comprising items with good discrimination (the ability to discriminate patients with different levels of health literacy) that range in difficulty (easy to hard). Health LiTT has been shown to be acceptable to patients, including those who are not sophisticated computer users (Hahn et al., 2014; Yost et al., 2010).

The validation of any measurement instrument is an ongoing process of accumulating evidence showing that the tool is measuring the intended construct and that the resulting scores behave as hypothesized (Crocker & Algina, 1986). Thus, the objective of this article is to add to this body of evidence by providing information on the convergent validity and reliability of a 10-item Health LiTT short form in a sample of community-dwelling older adults. Additionally, a cutoff score was identified, allowing for a dichotomous measure of adequate versus inadequate/marginal health literacy. Dichotomous classification provides a strategy to estimate the prevalence of the population at risk from low health literacy and can be used to stratify people for testing interventions.

## Methods

The data for this analysis were obtained from the Literacy and Cognitive Function among Older Adults (LitCog) study (Wolf et al., 2012). The primary objectives of LitCog were to investigate the relationship between health literacy and domains of cognitive function in older adults, and to determine how these factors predict one's ability to perform routine heath activities.

### Participants

English-speaking adults between ages 55 and 74 who received care at an ambulatory care clinic or at 1 of 3 federally qualified health centers in Chicago, IL, were recruited. The final LitCog sample included 832 participants who completed study procedures from August 2008 to November 2010. A subset of 137 participants completed the Health LiTT short form during the last year of the study (November 2009–November 2010). Complete data for the validation analyses were available for 133 of the subset of 137 participants who were administered the Health LiTT short form in the LitCog study.

### Procedures

Participants completed two interviews 7 to 10 days apart, each lasting roughly 2.5 hours and conducted by trained research assistants. The interviewers administered three commonly used health literacy measures and a comprehensive cognitive battery. Additional data collected during the interviews included demographic information, socioeconomic status, comorbidity, and performance on everyday health tasks (Wolf et al., 2012). At the end of the last day of testing during the last year of the LitCog study (November 2009–November 2010), the 137-patient subset also completed a 10-item Health LiTT short form via self-administration on a touchscreen laptop computer. Northwestern University Institutional Review Board approved the study.

### Measures

The three interviewer-administered measures of health literacy assessed in LitCog were the Test of Functional Health Literacy in Adults (TOFHLA) (Baker et al., 2000; Nurss et al., 1995; Parker et al., 1995), the Rapid Assessment of Adult Literacy in Medicine (REALM) (Davis et al., 1993), and the Newest Vital Sign (NVS) (Weiss et al., 2005). The TOFHLA measures reading comprehension of health-related text using a modified Cloze procedure in which respondents select a multiple-choice option that best fills in a missing word in a phrase. Numeracy is measured in the TOFHLA by showing the respondent a health-related prop (i.e., prescription label, appointment card) and then asking a question about the information in the prop. For the REALM, participants are presented with a list of health-related terms and asked to pronounce them. The score is based on the number of words pronounced correctly as judged by the interviewer. The NVS involves participants reviewing a nutrition label and answering questions assessing their document and quantitative skills. Higher scores on all three measures indicate better health literacy and cut scores have been established to place people into discrete categories of health literacy.

For this study, 10 Health LiTT items (four prose, three document, and three quantitative) were selected from the item bank to create a short form that spanned the range of item difficulty and covered diverse content. The 10-item short form takes about 5 to 7 minutes to complete via computer-assisted self-administration. IRT-based scores were determined by using item responses and item calibrations. The 10-item short form is scored on a T-score scale, which has a mean of 50 and standard deviation of 10 in the calibration sample (Hahn et al., 2011). Higher T-scores on the short form indicate better health literacy.

## Data Analysis

### Analyses of Validity and Reliability

There are numerous ways of assessing the validity of a measurement tool. In this study, convergent validity was assessed by comparing scores for the Health LiTT short form to scores for other, commonly used measures of health literacy. If Health LiTT is measuring health literacy—the intended construct—the short form scores should correlate strongly with scores of other measures. Due to violation of normality assessed via Shapiro-Wilk test (*p* < .05 for all measures) and the categorical nature of the TOFHLA, REALM, and NVS, Spearman correlations were used. The criterion for strong correlation was a Spearman correlation of −0.5 or greater (Cohen, 1988).

Known-groups validity, an indicator of how well Health LiTT scores discriminate groups of patients who are hypothesized to have different levels of health literacy, was also evaluated. Analysis of variance (ANOVA) was implemented where the known groups were categories of health literacy defined by the TOFHLA total score (inadequate, marginal, and adequate). Effect sizes were calculated for the known groups comparisons by dividing the mean Health LiTT score difference across known groups (i.e., inadequate vs. marginal, marginal vs. adequate) by the overall Health LiTT standard deviation of the sample. A positive association between health literacy scores and educational attainment has been well established (Baker et al., 2000; Baker et al., 2002a; Federman et al., 2009; Gazmararian et al., 1999; Kalichman & Rompa, 2000; Levinthal et al., 2008; Morrow et al., 2006; Williams et al., 1995; Wolf et al., 2012; Wolf et al., 2010); therefore, this expected association was evaluated for the Health LiTT short form with a Spearman correlation.

Finally, internal consistency reliability, as measured by Cronbach's alpha, was evaluated as an indication of the extent to which items that constitute the measure correlate with one another. A Cronbach's alpha of 0.7 or higher is considered sufficiently reliable for group-level applications, whereas an alpha of 0.9 or higher is sufficient for an individual-level applications (Nunnally & Bernstein, 1994). All analyses were performed with SAS version 9.2.

### Sensitivity and Specificity

The IRT calibrations for Health LiTT can produce a precise continuous score of health literacy. As health literacy measures are often used to classify people, to allow for greater flexibility of use, Health LiTT short form scores were compared to TOFHLA scores to determine sensitivity, specificity, predictive values, and the receiver-operating characteristic (ROC curve) to identify a cutoff between marginal/inadequate and adequate health literacy. The TOFHLA alone was used to establish an optimal cutoff that maximizes overall validity (i.e., high sensitivity and specificity) due to the similar scale and question structure as Health LiTT. Analyses were performed using the ROCR package in R (R Core Team, 2013; Sing et al., 2007).

## Results

### Descriptive Statistics

As shown in **Table 1**, the sample was older (by design), predominantly African American women, and fairly well educated. Descriptive statistics for all health literacy measures are summarized in **Table 2**. The mean T-score for the 10-item Health LiTT short form was 53.5 (*SD* = 8.3). Because the mean T-score in the calibration sample is 50 (*SD* = 10), these results indicate that this LitCog subsample has higher and slightly less variable Health LiTT scores compared to the calibration sample on which the IRT scaling was based. Health LiTT scores were positively associated with educational attainment (Spearman correlation rs = 0.63, *p* < .001).

### Associations of Health LiTT with Other Health Literacy Measures

Spearman correlations between Health LiTT and other health literacy measures were greater than the criterion of 0.5 and ranged from 0.52 for the correlation with TOFHLA numeracy to 0.69 for the correlation with the REALM (**Table 3**). Spearman correlations between the other measures of health literacy were also high and ranged from 0.54 for the REALM and TOFHLA numeracy to 0.81 for the REALM and TOFHLA reading. The correlations between the TOFHLA total score and the TOFHLA reading and numeracy components were 0.80 and 0.93, respectively, but these values are inflated due to overlap of the reading and numeracy scores in the total score. All correlations were significant at the *p* < .001 level.

**Figure 1.**
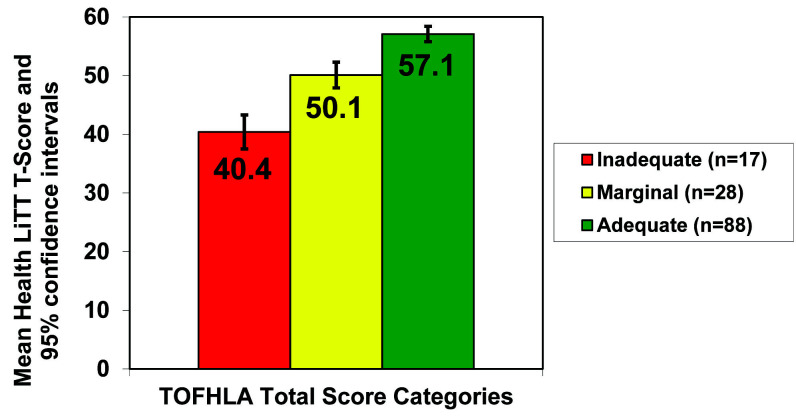
Known groups validity of a 10-item Health Literacy Assessment Using Talking Touchscreen Technology (Health LiTT) short form. Numbers within the columns indicate mean Health LiTT T-scores for those identified as having inadequate, marginal, and adequate health literacy according to the Test of Functional Health Literacy in Adults with identified confidence intervals.

For known-groups validity, participants were categorized into inadequate, marginal, and adequate based on the total TOFHLA scores. Most people (*n* = 88, 66.2%) were in the adequate category. Mean scores for the Health LiTT short form were significantly different across inadequate (mean = 40.4, *SD* = 5.9), marginal (mean = 50.1, *SD* = 7.7) and adequate (mean = 57.1, *SD* = 5.4) categories (F = 60.6; *p* < .001), demonstrating that Health LiTT discriminated well between health literacy levels (**Figure 1**). The overall ANOVA and each of the pairwise comparisons using unadjusted least squares means between the three groups were highly statistically significant (all *p* < .001). The difference in mean Health LiTT short form scores between inadequate and marginal categories was 9.7 points and corresponds to an effect size of 1.2. Between marginal and adequate categories, the difference in mean Health LiTT scores was 7 points, which corresponds to an effect size of 0.84. Cronbach's alpha was 0.73, which indicates that this 10-item Health LiTT short form has sufficient reliability to be used for group-level comparisons.

### Sensitivity and Specificity Analysis

**Figure 2.**
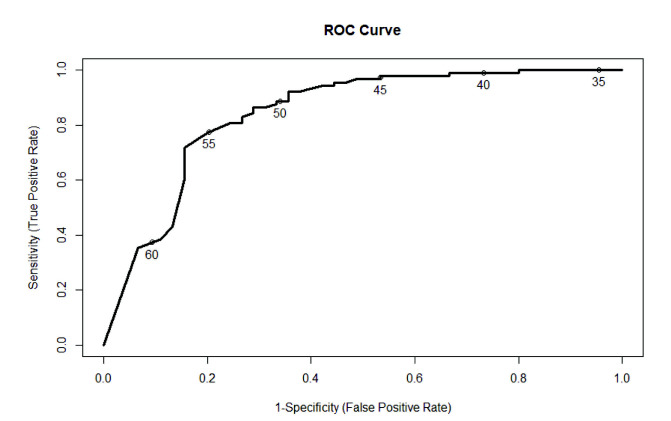
Receiver Operating Characteristic (ROC) curve for Health Literacy Assessment Using Talking Touchscreen Technology Short Form. Numbers on the ROC curve indicate possible cut-off points, with 55 demonstrating the best cut-point to maximize both sensitivity and specificity.

Although Health LiTT can be used to determine a continuous measure of health literacy, it is also useful to determine the best cut-point for distinguishing between adequate and marginal/inadequate health literacy. A Health LiTT score of 55 was determined as the best cutoff to maximize both sensitivity (77.2%) and specificity (80%) with an area under the ROC curve for predicting TOFHLA scores of 0.84. Positive predictive value was 88.3%, whereas negative predictive value was 64.3%. The optimum cutoff score for detecting adequate health literacy can also be determined visually from the ROC curve (**Figure 2**) as the score on the curve closest to the upper left corner of the plot.

## Discussion

This study adds to previous work on how the Health LiTT measurement system addresses the eight attributes recommended for multi-item measures of latent traits: (1) a conceptual and measurement model, (2) reliability, (3) validity, (4) responsiveness, (5) interpretability, (6) low respondent and administrative burden, (7) alternative forms, and (8) cultural and language adaptations (Aaronson et al., 2002). Prior work described the conceptual model and rigorous methodology for item development, measurement reliability, content validity, construct validity, state-of-the-science psychometric methods for calibration and interpretability, low respondent and administrative burden, and Spanish adaptations (Hahn et al., 2011; Yost et al., 2009; Yost et al., 2010). To that body of evidence, this study adds validity and reliability of a Health LiTT short form. The short form displays convergent validity with three other measures of health literacy with large effect sizes (Cohen, 1988) that can be interpreted as meaningful differences between health literacy levels rather than simply statistical significance due to sample size, as well as a positive association with education and good internal consistency reliability. These findings are bolstered by the fact that the sample evaluated here (mean age 62 years, range 55–74; 64% female; 34% non-Hispanic White; 33% with a high school or less education) is different in several respects from the sample with which the item bank was developed and calibrated (mean age 46 years, range 21–77; 50% female; 16% non-Hispanic White; 56% with a high school or less education) (Hahn et al., 2011). This suggests invariance in the psychometric properties of Health LiTT across samples. This study also established a useful cutoff score to classify adequate health literacy. Thus, Health LiTT provides alternative assessments of health literacy, in that users can obtain both a continuous or dichotomous measure (adequate vs. marginal/inadequate health literacy) depending on their individual clinical or research needs. This cutoff shows good sensitivity and specificity as compared with the TOFHLA, a gold-standard measure of health literacy, as well as sensitivity, specificity, and area under the curve (AUC) being comparable to standardized measures of health literacy. Health LiTT showed higher sensitivity than the NVS and a similar level to the REALM-Revised, with specificity and AUC values falling between these two measures (NVS sensitivity 72%, specificity 87%, AUC 0.88; REALM-Revised sensitivity 80.8, specificity 61.7, AUC 0.80) (Carpenter et al., 2014; Weiss et al., 2005).

There are different criteria for identifying optimal cutoff scores for a continuous measure. We chose to define the “optimal” cut-off as the score that maximized overall validity; that is, maximized both sensitivity and specificity and minimized both false positives and false negatives. Many screening tests accept lower specificity (i.e., more false positives) to achieve higher sensitivity, especially when the consequence of a false negative screen is severe, such as mammography screening for breast cancer. However, it is difficult to argue for selecting a cutoff that confers higher sensitivity and lower specificity (or vice versa) without knowing the specific intended use of the Health LiTT and being able to balance the consequences of false positives and negatives. For these reasons, we recommend a Health LiTT cut-off score of 55, which has approximately equal sensitivity and specificity when compared to the TOFHLA, as it will maximize the correct classification of those with and without marginal/inadequate health literacy.

Health LiTT provides even greater flexibility, as in principle a custom short form can be comprised of any of the items in the 82-item bank, allowing users to determine the type and difficulty level of questions they may need for a given project. Health LiTT shows great promise in providing rapid assessment of health literacy and appears to have good psychometric properties that are agnostic to the study sample and assessment setting.

## Study Limitation and Future Directions

One limitation to this study is the inability at this time to employ predictive validity analyses in relation to constructs associated with health literacy, such as health-related quality of life or health outcomes. Establishing predictive validity of Health LiTT will be an important area for future research with continued validation in different clinical settings and populations providing greater evidence of its utility and psychometric properties. Health LiTT is free to use for clinical practice and nonprofit clinical research and can be accessed via www.healthlitt.org.

## Figures and Tables

**Table 1:** Participant Characteristics (*N* = 133^a^)

**Characteristic**	***n* (%)**

Mean age, years (*SD*)	62.5 (5.2)

Female	85 (63.9)

Race/ethnicity	
Non-Hispanic Black	73 (54.9)
Non-Hispanic White	45 (33.8)
Non-Hispanic Asian	3 (2.3)
Hispanic (race not specified)	6 (4.5)
Other^b^	6 (4.5)

Education	
Less than high school	14 (10.5)
High school graduate or GED	30 (22.6)
Some college/technical school	34 (25.6)
College graduate	23 (17.3)
Graduate degree	32 (24.1)

Note. GED = General Educational Development.

a Total participants were 137, but 133 represents the final sample.

b Multiple responses that included biracial, bicultural, and Native American.

**Table 2:** Mean Scores and Score Ranges of Health Literacy Measures (*N* = 133^a^)

**Measure**	**M (*SD*)**	**Range**
10-item Health LiTT short form	53.5 (8.3)	31.8–62.2
TOFHLA numeracy	31.8 (7.5)	3–48
TOFHLA reading	44.3 (8.6)	0–50
TOFHLA total	76.2 (14.1)	18–97
REALM	59.3 (11.4)	6–66
NVS	2.8 (2)	0–6

Note. Health LiTT = Health Literacy Assessment Using Talking Touchscreen Technology; NVS = Newest Vital Sign; REALM = Rapid Estimate of Adult Literacy in Medicine; TOFHLA = Test of Functional Health Literacy in Adults.

a Total participants were 137, but 133 represents the final sample.

**Table 3:** Spearman Correlations of Health Literacy Measures (*N* = 133^a^)

		**Spearman correlation, *r****_s_*			
**Measure**	**Health LiTT short form**	**REALM**	**NVS**	**TOFHLA total**	**TOFHLA reading**
REALM	0.69	-	-	-	-
NVS	0.56	0.69	-	-	-
TOFHLA total	0.65	0.72	0.67	-	-
TOFHLA reading	0.67	0.81	0.64	0.80	-
TOFHLA numeracy	0.52	0.54	0.58	0.92	0.57

Note. Health LiTT = Health Literacy Assessment Using Talking Touchscreen Technology; NVS = Newest Vital Sign; REALM = Rapid Estimate of Adult Literacy in Medicine; TOFHLA = Test of Functional Health Literacy in Adults.

a Total participants were 137, but 133 represents the final sample.
